# Supplementary material for: Handedness-dependent quasiparticle interference in the two enantiomers of the topological chiral semimetal PdGa
Source: Nat Commun. 2020 Jul 14;11:3507. doi: 10.1038/s41467-020-17261-x (PMC7360625; doi:10.1038/s41467-020-17261-x)
Supplement: Supplementary file 1 — Supplementary Information [file 41467_2020_17261_MOESM1_ESM.pdf]

Supplementary Information for:

Handedness-dependent quasiparticle interference in the two enantiomers of the topological chiral semimetal PdGa

Paolo Sessi et al.

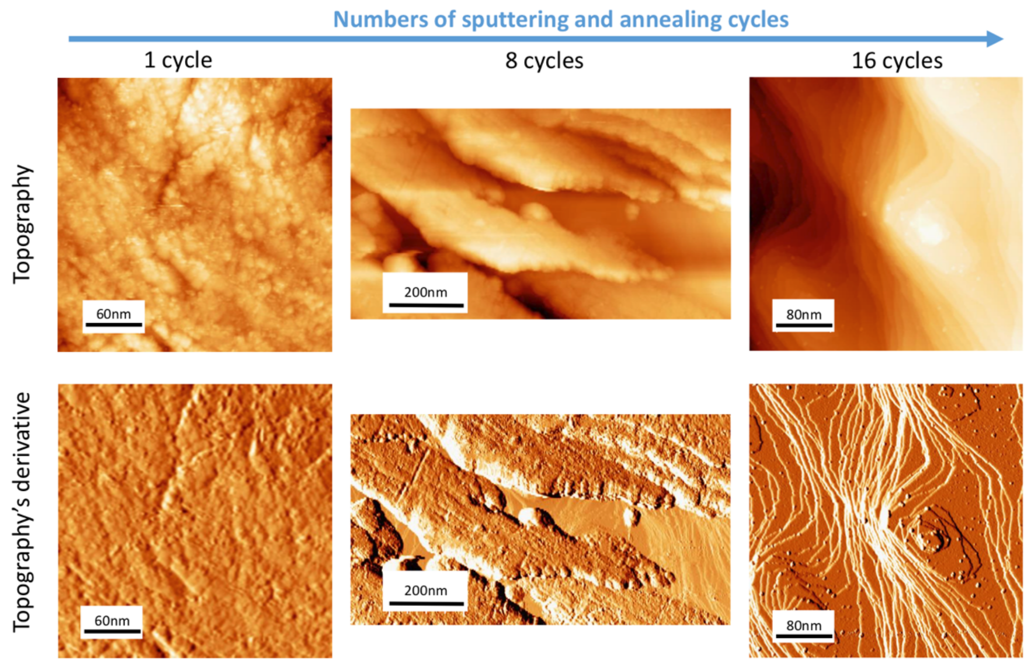

Supplementary Figure 1| Sample preparation. Top row: Sample topography as a function of the number of sputtering and annealing cycles. Clean areas start to become visible after 8 cycles. Bottom row: derivate of the images reported in the top row, which allows to better identify the evolution of the surface roughness.

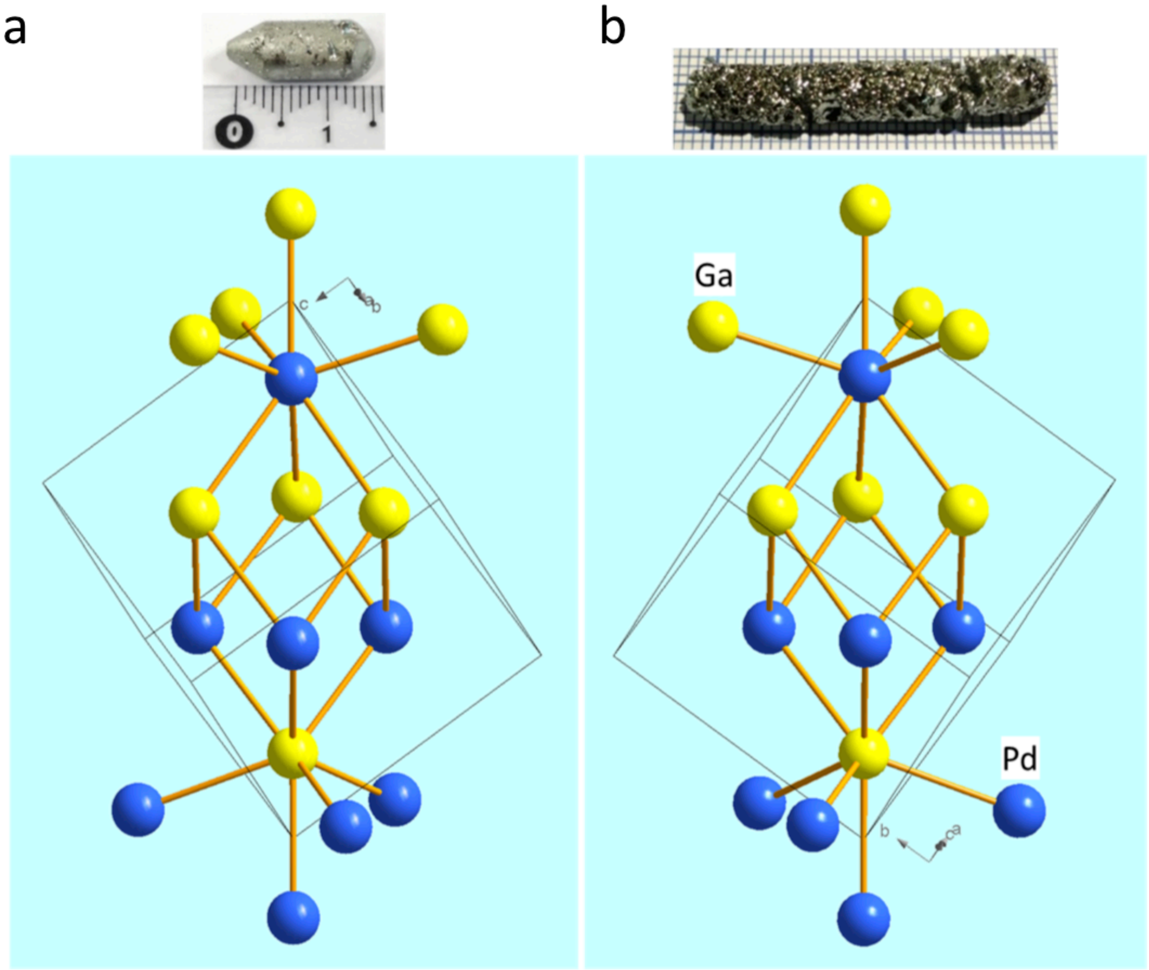

Supplementary Figure 2 | Crystal structure. Picture of PdGa single crystals and corresponding structural unit. The Wyckoff positions obtained from the diffraction experiments are (a): [Pd at x = 0.14246(4), Ga at x = 0.84301(6)] and (b): [Pd at x = 0.85758(3), Ga at x = 0.15694(5)]. Positions of atoms in the asymmetric unit were assigned according to ref. 7.

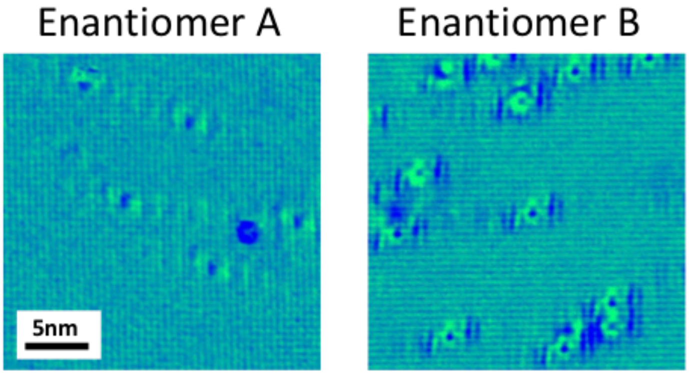

Supplementary Figure 3 | Crystal defects. Perturbation pattern developing around native defects in the two enantiomers.

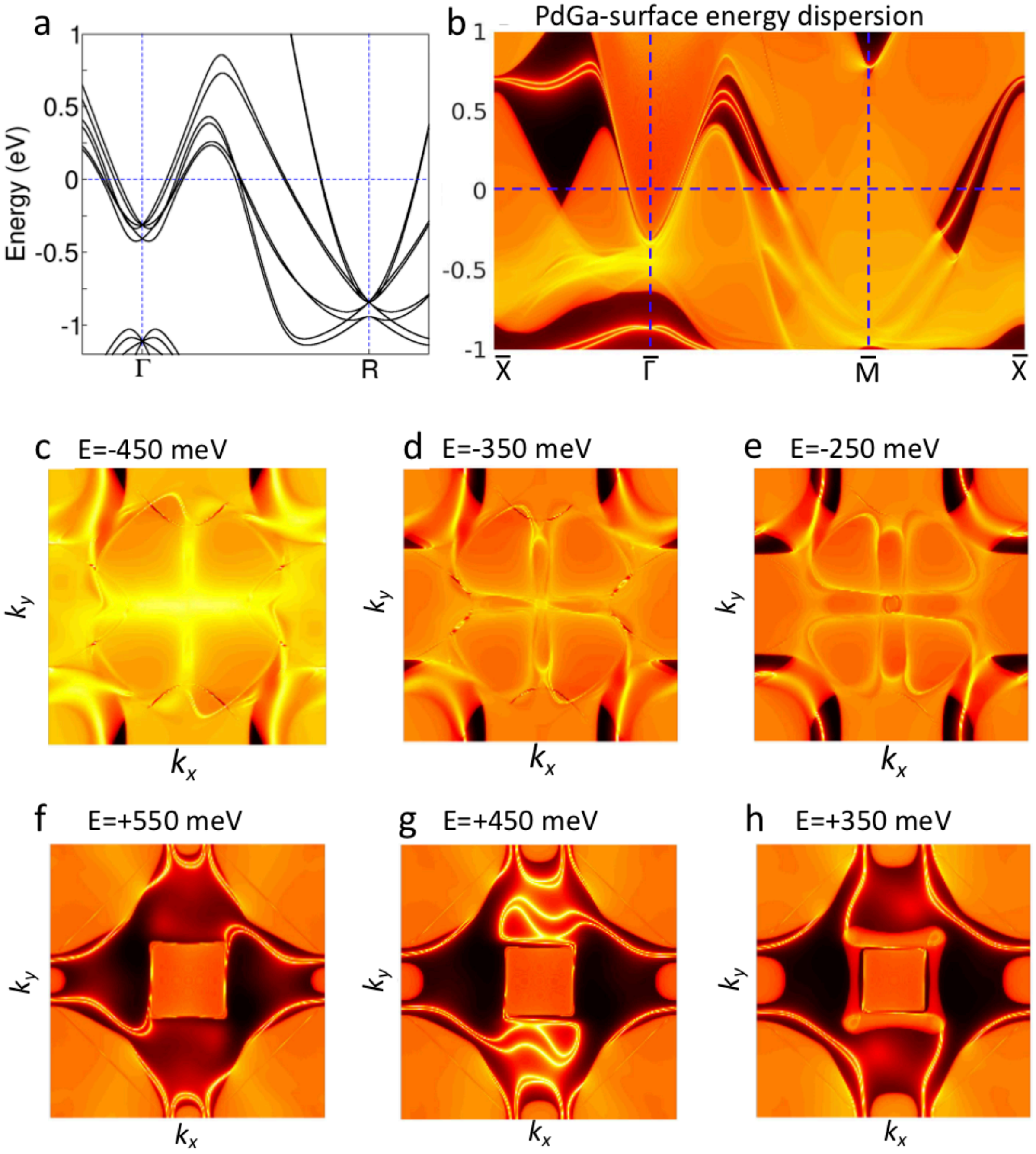

Supplementary Figure 4 | Bulk and surface states of PdGa from DFT calculations. a Bulk energy dispersion along Γ-R. **b** Surface energy dispersion along high symmetry lines. **c-h** Constant energy contours of surface states.

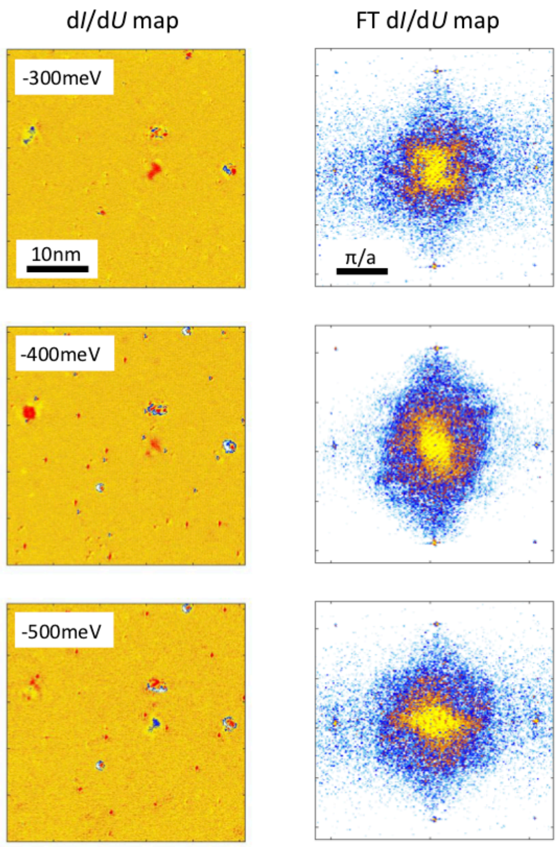

Supplementary Figure 5 | Quasiparticle interference for occupied states. As described in Supplementary Note 1, Fermi arcs are strongly overlapping with bulk projected states at energies below the Fermi level. This scenario complicates the experimental detection of topological states. In quasiparticle interference experiments, this results in a continuum of possible scattering vectors, where not any clear feature is visible in the FT-dI/dU maps.

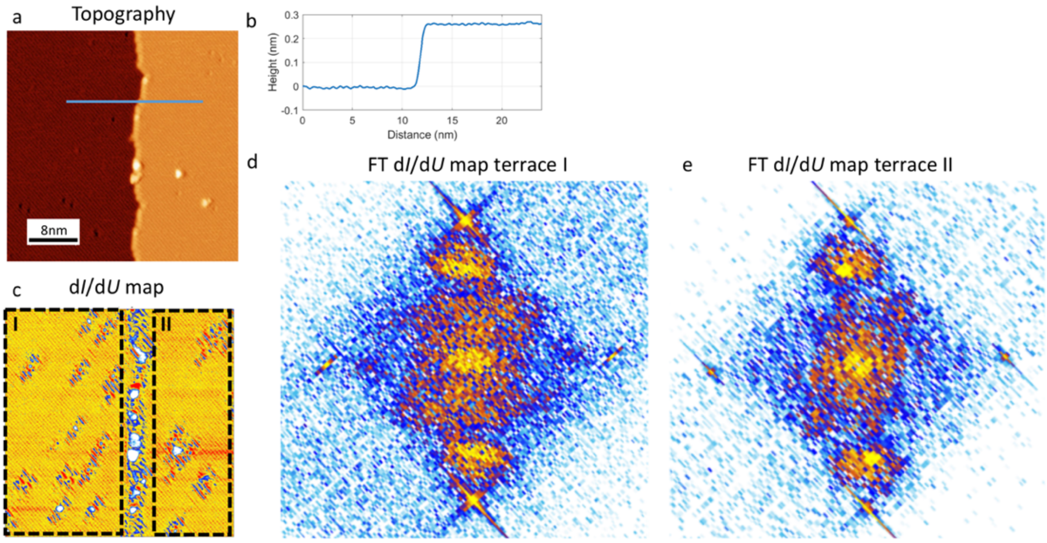

Supplementary Figure 6 | Quasiparticle interference on adjacent terraces. a Topographic image of two adjacent terraces. As visualised by the line profile reported in **b**, the difference in hight correspond to approximately 2.5 Å, i.e. half unit cell. **c** dI/dU maps; **d** and **e** report the Fourier transformed dI/dU maps obtained on terrace I and II, respectively. Their simultaneous investigation also allows to exclude changes in the tip apex configuration. Although the reduced dimension and the consequently low number of scattering centers reduces the resolution of the Fourier transformation, both terraces show the same chirality. This is consistent with PdGa belonging to space group $P2_13$: the termination of the lower terrace is the same as the upper one, just rotated by 180° and translated.

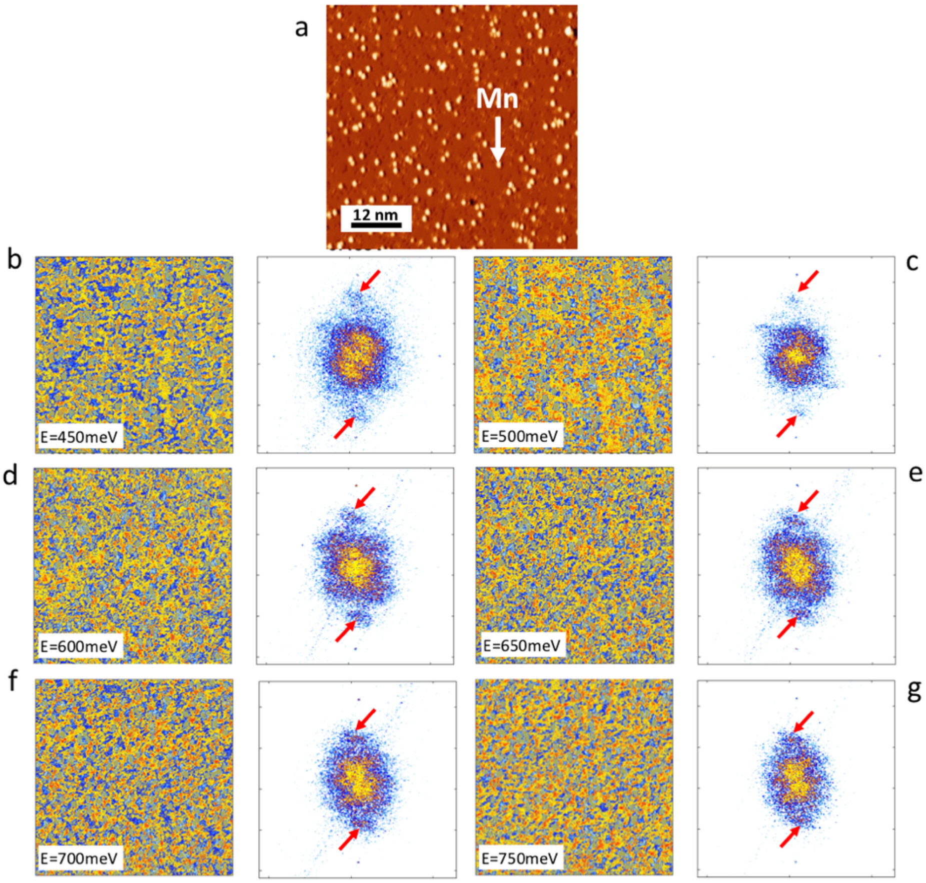

Supplementary Figure 7 | Mn doped surface. To further corroborate the topological origin of our observations, Mn adatoms have been intentionally evaporated onto the PdGa(001) surface using an e-beam evaporator. Single atoms were deposited directly into the microscope by keeping the sample at a temperature $T \simeq 15$ K. As shown in Supplementary **a**, Mn adatoms appear as bright protrusions (indicated by white arrow) with an apparent height of approximately 1 Å. Their presence significantly increases the disorder, enhancing the background in the Fourier transformations, as visible in panels **b**-**g**. However, large **q** scattering events originating by surface Fermi arcs [A(A') in the main text] are still visible (highlighted by red arrows), providing compelling evidence of their robustness associated to their topological origin.

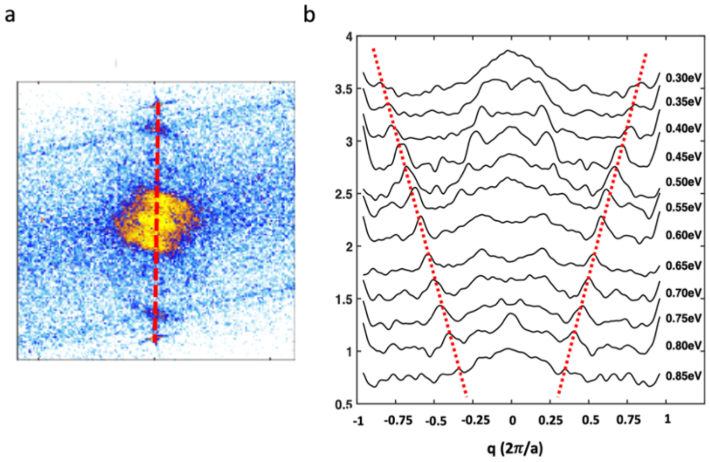

Supplementary Figure 8 | Energy dispersion. Analysis procedure for scattering events between opposite topological Fermi arcs. Intensity profiles have been taken, for all energies, along the dashed red line passing through the center of the FT-dI/dU map, as illustrated in **a**. Data obtained at every energy are reported in **b**. Each line has been vertically shifted for clarity. Dashed lines highlight the dispersion. The error bars reported in the main text correspond, for each energy, to the full width at half maximum of a gaussian fit to the intensity peak.

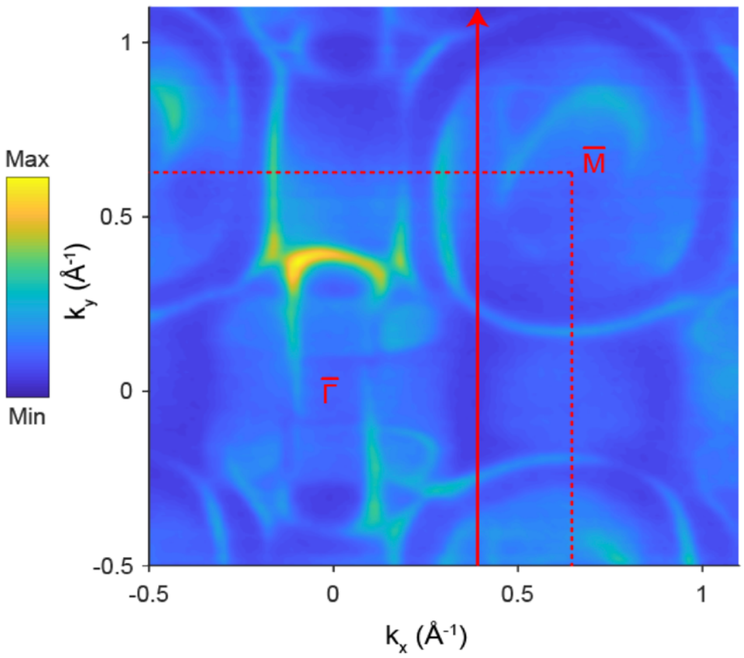

Supplementary Figure 9 | ARPES measurements. The measurement was performed with a photon energy of 60 eV and linear-horizontal polarization. The solid red arrow indicates the momentum position of the line-cut shown in Fig. 5b of the main manuscript. The dashed red line indicates the boundary of the surface Brillouin zone.

Supplementary Note 1. Emergence of chiral fermions and evolution of the constant energy cuts

The chiral fermions can be generally described by a unified form of $H = \chi \mathbf{k} \cdot \mathbf{S}$, with $\chi = \pm 1$ representing the chirality and the pseudospin matrix following $[S_i, S_j] = i\varepsilon_{ijk}S_k$[1]. Owing to combined time reversal symmetry, crystal twofold screw rotation, diagonal cubic threefold rotation, and the non-symmorphic symmetries, the fourfold and sixfold fermions locate at Γ and R point, respectively, near the Fermi level. The fourfold degeneracy at Γ point is a spin-3/2 excitation with a 4 by 4 pseudospin matrix and Chern number ±4. The sixfold degeneracy at R points is constructed by double spin-1 Weyl fermions and a doubly degenerated quadratic bands. Therefore, it can be described by a 3 by 3 pseudospin matrix with Chern number ∓2, making the whole system to follow the "no-go" theorem. Since there is not any symmetry connecting the R and Γ points, the two multifold chiral fermions are allowed to lie at different energies. As for PdGa, the energy difference is around 0.5 eV, with a non-trivial energy window ranging from -1.0 to 0.8 eV, where surface Fermi arcs connect the bulk states around R and Γ points, as presented in Supplementary Figure 4.

The density functional theory calculations were performed by using the full-potential local-orbital code (FPLO) with localized atomic basis and full potential treatment [2]. The exchange and correlation energy were considered at the generalized gradient approximation level (GGA) [3]. To construct the tight binding model Hamiltonian, we projected the Bloch wave functions onto atomic-like Wannier functions. With tight binding model Hamiltonian, we calculated the surface states using half-infinite open boundary condition and Green's function method [4,5]. The spin selected quasiparticle interference (QPI) patterns were calculated based on the Fourier transformed surface local density of states from the Green's function.

For occupied states, the topological Fermi arcs strongly overlap with bulk projected trivial states, severely complicating their identification, as visible in Supplementary Figure 4, c to e. This is not the case for unoccupied states, where Fermi arcs become strongly decoupled from the bulk electronic structure (see Supplementary Figure 4, f to h), a scenario favoring their unambiguous experimental detection. The band structure calculations and related constant energy cuts allow to highlight two distinct features which make topological chiral semimetals remarkably different from Weyl semimetals, namely: (i) band crossing locked at high symmetry points (panel a) and Fermi arcs spanning the entire Brillouin zone (panels c to h).

Supplementary Note 2: Consistency between bulk structural chirality and perturbation patterns developing around defects

The structural chirality of the two PdGa single crystals enantiomers has been analyzed by rigorous single crystal X-ray diffraction experiments. Small single crystal pieces were picked from different parts of the grown crystals to truly reflect the structural properties of the entire sample. The selected crystallites were mounted on Kapton loops with aid of a trace of Apiezon H grease and used in diffraction experiments on a Rigaku AFC7 four-circle diffractometer with a Saturn 724+ CCD-detector applying graphite-monochromatized Mo-Kα radiation. All selected crystals were fully characterized and complete crystallographic information with final results are compiled in standard .cif format and may be retrieved at (https://www.ccdc.cam.ac.uk/structures/) providing deposition numbers CSD-1999938 to CSD-1999942. The determination of the absolute structure via refinement of Flack's parameter confirmed single domain crystals without any significant contribution from twinning by inversion [6]. This fully confirms the proper

assignment of handedness as expected from crystal growth as shown in Supplementary Figure 2 for a right handed (or A-form crystal in ref. 7) and b left-handed (or B-form crystal in ref. 7). As shown in Supplementary Figure 3, the perturbation pattern developing around all native defects is consistent with the bulk characterization of the structural handedness.

Supplementary References:

1. Bradlyn, B. *et al.* Beyond Dirac and Weyl fermions: Unconventional quasiparticles in conventional crystals. *Science* **353**, 5037 (2016).
2. Koepernik, K. & Eschrig, H. Full-potential nonorthogonal local-orbital minimum-basis band-structure scheme. *Phys. Rev. B* **59**, 1743 (1999).
3. Perdew, J. P., Burke, K., & Ernzerhof, M. Generalized Gradient Approximation Made Simple. *Phys. Rev. Lett.* **77**, 3865 (1996).
4. Sancho, M. P. L., Sancho, J. M. L & Rubio, J. Quick iterative scheme for the calculation of transfer matrices. *Journal of Physics F: Metal Physics* **14**, 1205 (1984).
5. Sancho, M. P. L. *et al.* Highly convergent schemes for the calculation of bulk and surface Green functions. *Journal of Physics F: Metal Physics* **15**, 851 (1985).
6. Sheldrick, G. M. Crystal structure refinement with SHELXL. *Acta Crystallographica Section C* **71**, 3 (2015).
7. Spence, J. C. H. *et al.* On the minimum number of beams needed to distinguish enantiomorphs in X-ray and electron diffraction. *Acta Crystallographica Section A* **50**, 647 (1994).
