# Supplementary material for: Handedness-dependent quasiparticle interference in the two enantiomers of the topological chiral semimetal PdGa
Source: Nat Commun. 2020 Jul 14;11:3507. doi: 10.1038/s41467-020-17261-x (PMC7360625; doi:10.1038/s41467-020-17261-x)
Supplement: Supplementary file 2 — Peer Review File [file 41467_2020_17261_MOESM2_ESM.pdf]

Editorial Note: This manuscript has been previously reviewed at another journal that is not operating a transparent peer review scheme. The manuscript was considered suitable for publication without further review at Nature Communications .
